# Melatonin suppresses hepatocellular carcinoma progression via lncRNA-CPS1-IT-mediated HIF-1α inactivation

**DOI:** 10.18632/oncotarget.19316

**Published:** 2017-07-18

**Authors:** Tong-Hong Wang, Chi-Hao Wu, Chau-Ting Yeh, Shih-Chi Su, Shih-Min Hsia, Kung-Hao Liang, Chin-Chuan Chen, Chuen Hsueh, Chi-Yuan Chen

**Affiliations:** ^1^ Tissue Bank, Chang Gung Memorial Hospital, Tao-Yuan, Taiwan; ^2^ Graduate Institute of Health Industry Technology and Research Center for Industry of Human Ecology, College of Human Ecology, Chang Gung University of Science and Technology, Tao-Yuan, Taiwan; ^3^ Liver Research Center, Department of Hepato-Gastroenterology, Chang Gung Memorial Hospital, Tao-Yuan, Taiwan; ^4^ Department of Human Development and Family Studies, National Taiwan Normal University, Taipei, Taiwan; ^5^ Whole-Genome Research Core Laboratory of Human Diseases, Chang Gung Memorial Hospital, Keelung, Taiwan; ^6^ School of Nutrition and Health Sciences, College of Nutrition, Taipei Medical University, Taipei, Taiwan; ^7^ Graduate Institute of Natural Products, Chang Gung University, Tao-Yuan, Taiwan; ^8^ Department of Anatomic Pathology, Chang Gung Memorial Hospital, Chang Gung University School of Medicine, Tao-Yuan, Taiwan

**Keywords:** melatonin, hepatocellular carcinoma, epithelial-mesenchymal transition, lncRNA-CPS1-IT1, HIF-1α

## Abstract

Melatonin is the primary pineal hormone that relays light/dark cycle information to the circadian system. It was recently reported to exert intrinsic antitumor activity in various cancers. However, the regulatory mechanisms underlying the antitumor activity of melatonin are poorly understood. Moreover, a limited number of studies have addressed the role of melatonin in hepatocellular carcinoma (HCC), a major life-threatening malignancy in both sexes in Taiwan. In this study, we investigated the antitumor effects of melatonin in HCC and explored the regulatory mechanisms underlying these effects. We observed that melatonin significantly inhibited the proliferation, migration, and invasion of HCC cells and significantly induced the expression of the transcription factor FOXA2 in HCC cells. This increase in FOXA2 expression resulted in upregulation of lncRNA-CPS1 intronic transcript 1 (CPS1-IT1), which reduced HIF-1α activity and consequently resulted in the suppression of epithelial-mesenchymal transition (EMT) progression and HCC metastasis. Furthermore, the results of the *in vivo* experiments confirmed that melatonin exerts tumor suppressive effects by reducing tumor growth. In conclusion, our findings suggested that melatonin inhibited HCC progression by reducing lncRNA-CPS1-IT1-mediated EMT suppression and indicated that melatonin could be a promising treatment for HCC.

## INTRODUCTION

Hepatocellular carcinoma (HCC) is the fifth most common cancer and ranks third among cancer-related deaths worldwide. HCC causes approximately 7,000 deaths annually and ranks second among the most deadly cancers in Taiwan [1–3]. Because HCC does not elicit obvious symptoms during its early stages, most individuals are diagnosed with this disease during its later stages, which results in poor patient prognoses. Currently, the first-line treatment for HCC is surgical resection. Patients who either are unable to undergo surgical resection or have metastatic cancer are treated with chemotherapy or radiation therapy. Unfortunately, the therapeutic outcomes for these patients are usually poor because the incidence of multidrug resistance is high among patients with HCC [4–6]. Even sorafenib, which is currently the most effective HCC-specific chemotherapeutic drug available, only extends the lifespans of affected patients by an average of three months [6, 7]. Furthermore, most anti-HCC drugs cause severe side effects that significantly interfere with the patient’s quality of life. Given the limitations of these current therapies, developing more effective therapeutic drugs with fewer or minimal side effects remains an important focus of HCC research.

Melatonin is a hormone that is primarily secreted by the pineal gland and relays light/dark cycle information to the circadian system [8, 9]. Melatonin is also secreted by other types of cells such as liver cells and villous cells in the large intestine [10, 11]. Melatonin circulates throughout the bloodstream and binds to melatonin receptors (MT1, MT2, and MT3) to regulate blood pressure and the heart rate [9]. The properties of melatonin and its derivatives are well-characterized and enable these molecules to function in maintaining circadian and seasonal rhythms as well as exert antioxidant activities to reduce cell damage by directly reacting with and reducing oxidized metabolites [12–14]. Moreover, melatonin has been reported to function in several other processes, including immune system regulation [15, 16], mitochondrial activity modulation [17, 18], cell death and autophagy regulation [19–21], and intrinsic antitumoral activity [22–24].

Recent studies on breast, lung, and colorectal cancer have shown that melatonin inhibits cancer cell growth, migration, and vascular invasion by blocking signal transduction pathways. Some of these pathways are involved in regulating the activation of matrix metalloproteinases (MMPs), hypoxia-inducible factor-1/signal transducer and activator of transcription 3 (HIF-1/STAT3), and molecules associated with nuclear factor-κB (NF-κB) [25–28]. Moreover, some clinical trials have demonstrated that melatonin therapy combined with other therapies can significantly extend the survival of patients with cancer, which highlights the potential usefulness of melatonin as an anti-cancer treatment [29]. Most anti-cancer drugs cause severe side effects; however, melatonin has not been reported to induce toxicity in human patients to date. This finding suggests that melatonin is an effective anti-cancer treatment agent that does not interfere with the patient’s quality of life. An increasing number of studies have evaluated the anti-cancer effects of melatonin; however, there are few reports describing the effects of melatonin in HCC, which is a life-threatening malignancy that affects both sexes in Taiwan. Further investigations are necessary to elucidate the mechanisms underlying the effects of melatonin in HCC.

Previous studies investigating the mechanism by which melatonin suppresses cancer progression have mainly focused on how melatonin affects protein expression levels. However, recent studies have confirmed that non-coding RNAs (ncRNAs) such as microRNAs (miRNAs) and long non-coding RNAs (lncRNAs) play an important role in regulating cell physiology [30–33]. lncRNAs are ncRNAs ranging from 200 bases to 100 kb in length and comprise ribosomal RNAs, transcribed pseudogenes, mRNA-like transcripts, and intronic transcripts. Most lncRNAs exhibit tissue-specific expression patterns and participate in regulating different aspects of gene expression, including chromatin reprogramming, transcriptional processing, and microRNA sponging, all of which modulate critical physiological functions [30, 31]. Dysregulation of lncRNA is correlated with the development and progression of many human diseases, including cancer, Alzheimer’s disease, and heart disease [34–38]. In this study, we comprehensively evaluated the mechanisms underlying the anti-HCC activity of melatonin. Moreover, we investigated whether melatonin is involved in regulating ncRNAs, their regulatory networks and their downstream targets in HCC.

## RESULTS

### Melatonin inhibited HCC proliferation, migration and invasion

**Figure 1:**
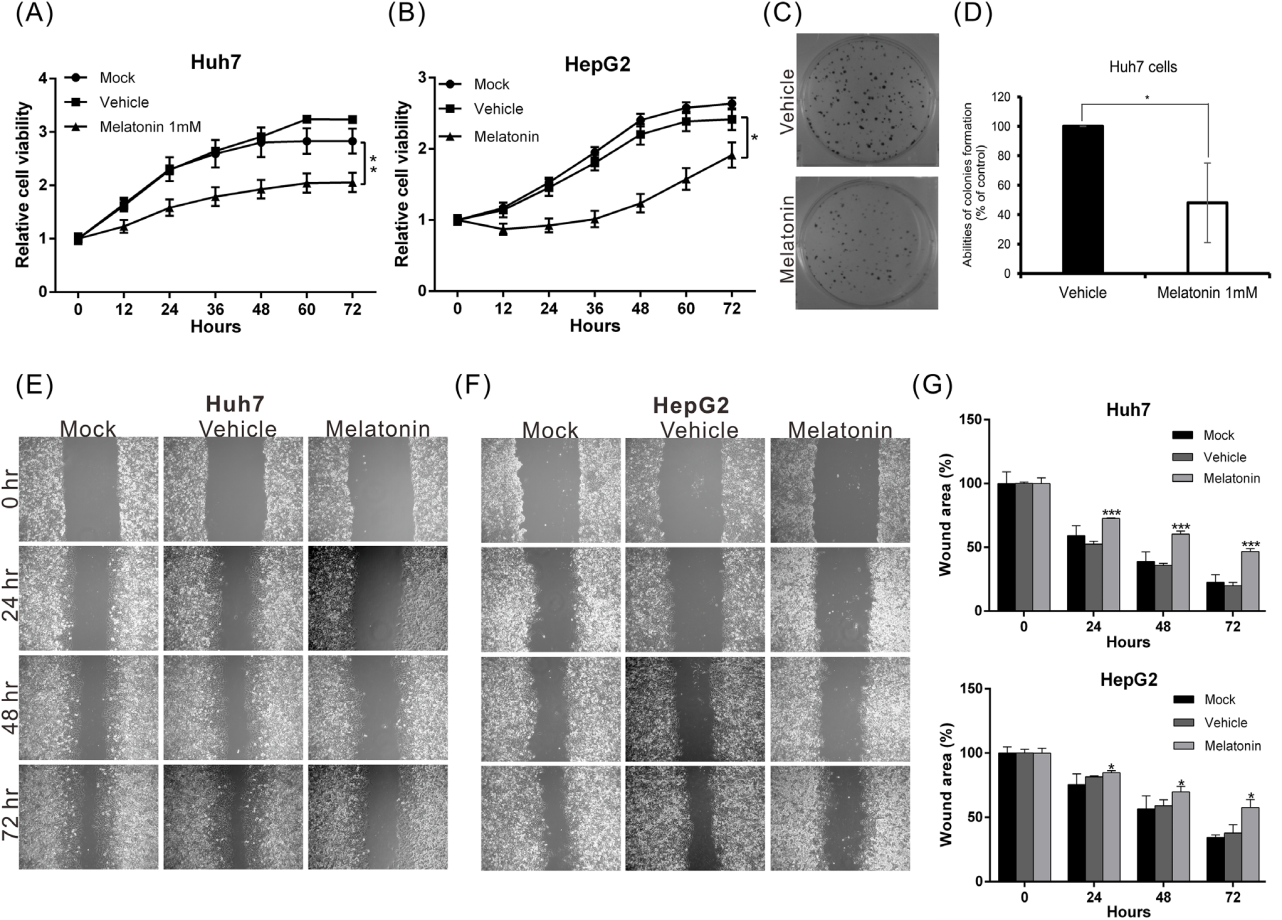
Melatonin suppressed HCC proliferation and migration *in vitro* **(A, B)** The proliferative capacities of Huh7 and HepG2 cells were monitored at the indicated time points using an xCELLigence real-time cell analyzer. Melatonin significantly suppressed the proliferative capacities of both cell lines. p < 0.01 (**). **(C, D)** The colony-forming ability was analyzed in cells in the presence or absence of melatonin for 12 days. The histogram shows that melatonin significantly inhibited the colony-forming ability of Huh7 cells. **(E, F)** The results of the wound-healing assay were compared between melatonin- and vehicle-treated Huh7 and HepG2 cells. Melatonin reduced the wound-healing ability of both cell lines. The quantitative wound-healing assay results are shown in **(G)**. p < 0.05 (*), p < 0.001 (***).

To investigate the biological role of melatonin in HCC progression, we treated Huh7 and HepG2 cells with different concentrations of melatonin and monitored the resultant proliferation rates using an xCELLigence real-time cell analyzer. The results revealed that melatonin suppressed cell proliferation in a dose-dependent manner beginning at a concentration of 0.1 mM. The half maximal inhibitory dose (ID_50_) for melatonin was 1 mM (data not shown). The proliferative ability of HepG2 and Huh7 cells treated with 1 mM melatonin for 48 hr was reduced by 34% and 44%, respectively, compared to corresponding cells treated with vehicle alone (Figure 1A, 1B). Similar results were observed in the colony formation assay (Figure 1C, 1D), wherein the colony-forming capacity was suppressed by 52% in Huh7 cells treated with 1 mM melatonin compared to Huh7 cells treated with vehicle.

**Figure 2:**
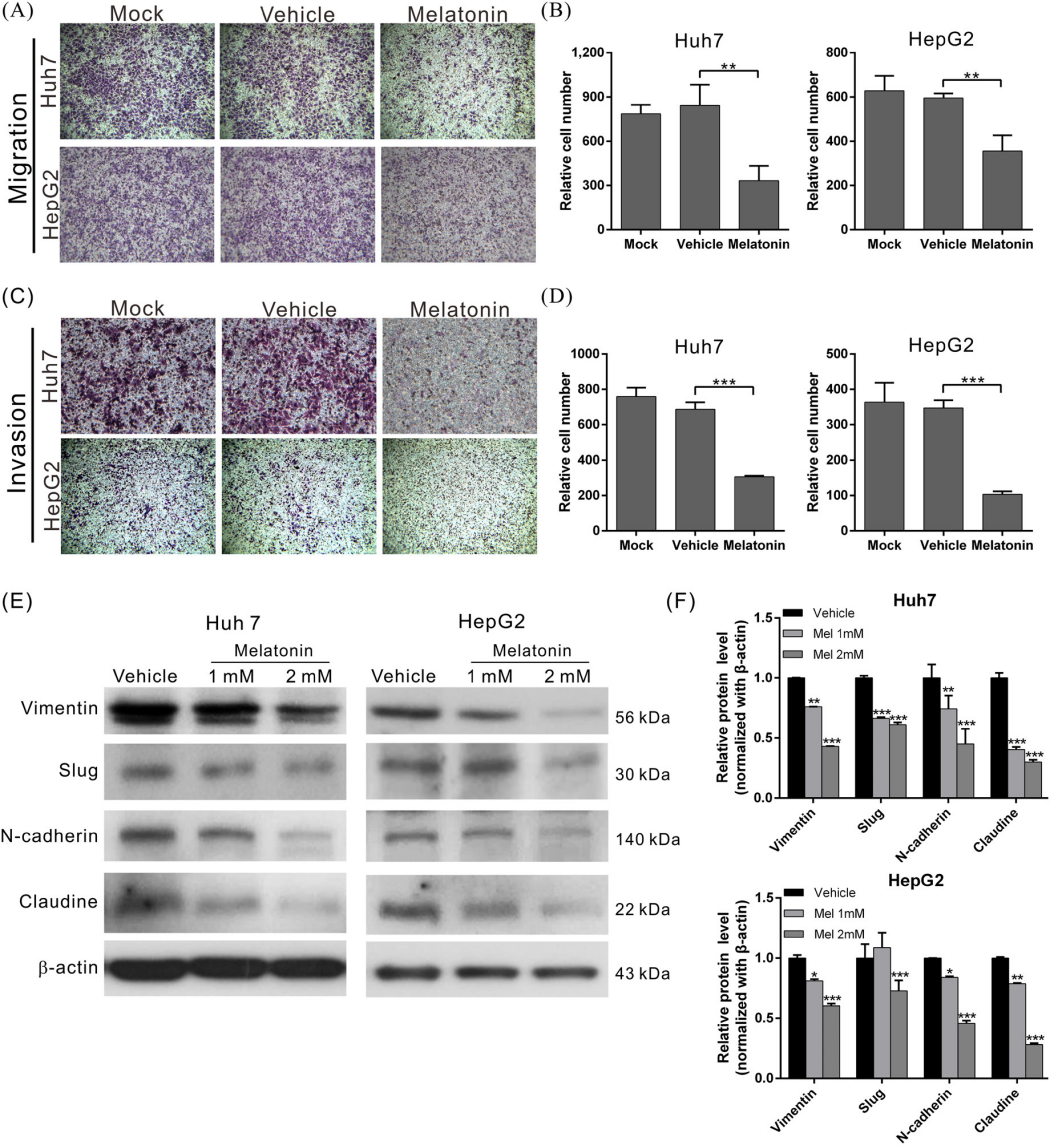
Melatonin suppressed HCC cell migration and invasion by inhibiting EMT **(A)** The migration abilities of Huh7 and HepG2 cells treated in the presence or absence of melatonin for 24 hr were compared using a transwell assay. Melatonin significantly reduced the migratory ability in the indicated cell lines. The quantitative cell migration assay results are shown in **(B)**. p < 0.01 (**). **(C)** Invasion assays were performed using Matrigel-coated polyethylene terephthalate membrane inserts. Five different fields (200× magnification) were imaged to quantify the numbers of migrating or invading cells. The quantitative cell invasion assay results are shown in **(D)**. p < 0.001 (***). **(E)** Western blotting analysis of the expression of EMT-related proteins in the indicated cells treated with either melatonin or vehicle. β-actin served as an internal control. Melatonin significantly reduced the expression levels of EMT-related proteins in Huh7 and HepG2 cells. The quantitative western blotting results are shown in **(F)** p < 0.05 (*), p < 0.01 (**), p < 0.001 (***).

To elucidate the effects of melatonin on tumor metastasis, we analyzed the migration and invasion capacities using wound-healing and transwell assays as appropriate. The migratory abilities of both melatonin-treated cell lines were significantly reduced compared to those of the corresponding vehicle-treated cells (Figures 1E-1G and 2A-2B). Similar results were observed in the cell invasion assay; the invasion capacities of both melatonin-treated cell lines were reduced by approximately 56% and 70%, respectively, compared to those of the corresponding vehicle-treated cell lines (Figure 2C, 2D). These results indicate that melatonin exerts tumor suppressive effects by repressing HCC cell proliferation, migration and invasion.

### Melatonin suppressed epithelial-mesenchymal transition in HCC cells

Epithelial-mesenchymal transition (EMT) is a major process associated with vascular invasion that results in intra- or extra-metastasis [39]. To evaluate the regulatory effects of melatonin on EMT, we treated Huh7 and HepG2 cells with melatonin for 24 hr and analyzed the expression patterns of several EMT-related proteins, including N-cadherin, vimentin, Slug, and claudin, in Huh7 and HepG2 cells using western blotting. The results revealed that melatonin significantly reduced the expression levels of the abovementioned EMT-related proteins in both cell lines in a dose-dependent manner (Figure 2E-2F), which suggested that melatonin exerts its anti-HCC activities by inhibiting EMT.

### Melatonin suppressed EMT via lncRNA-CPS1-IT1-mediated HIF-1α inactivation

**Figure 3:**
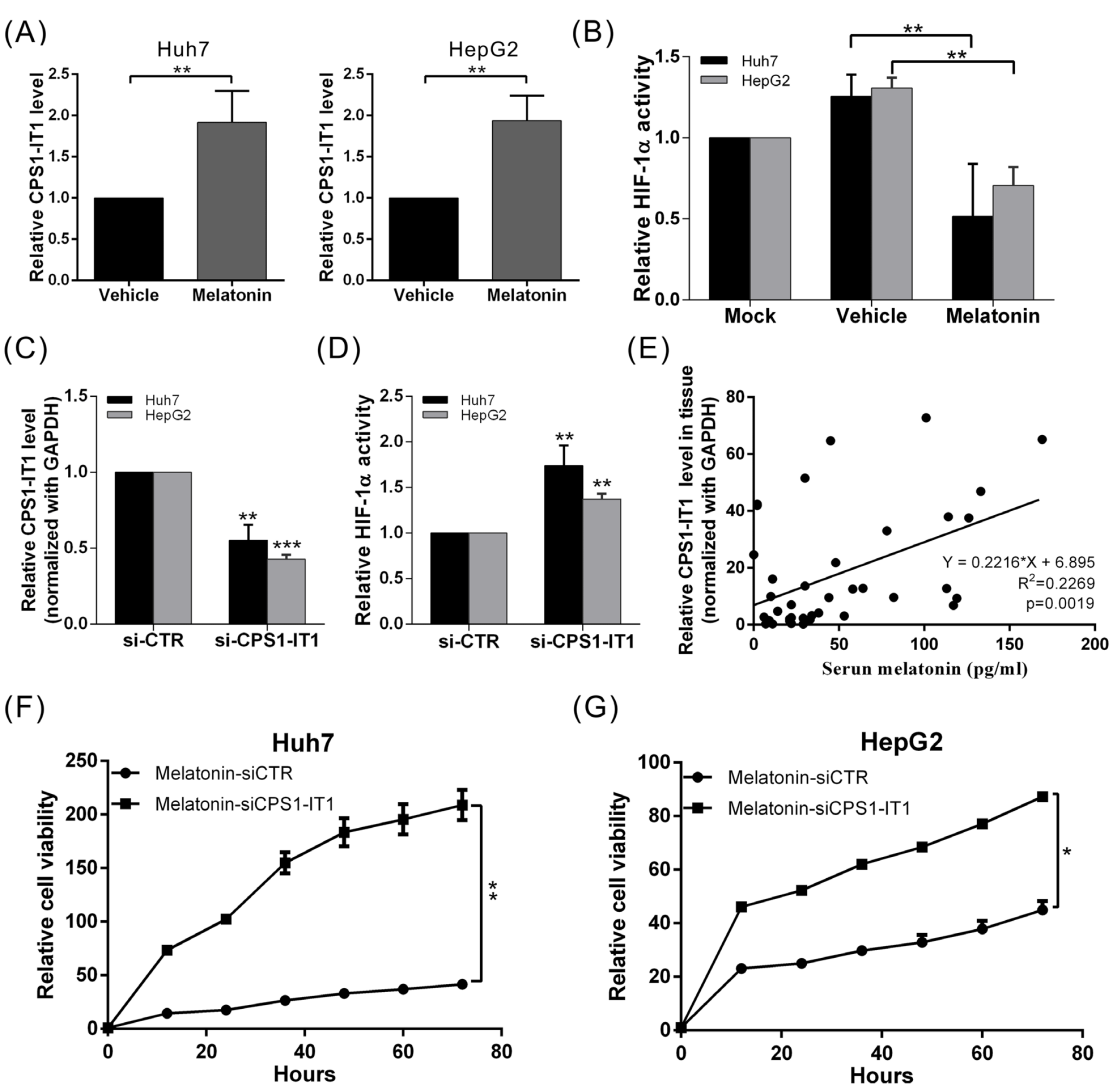
Melatonin inhibited HCC progression via CPS1-IT1-mediated HIF-1α inactivation **(A)** Huh7 and HepG2 cells were treated with melatonin for 24 hr, and CPS1-IT1 expression was analyzed using quantitative real-time RT-PCR, which showed that melatonin significantly induced CPS1-IT1 expression. **(B)** HIF-1α activity was analyzed in cells treated in the presence or absence of melatonin. Melatonin suppressed HIF-1α activation, and this effect was reversed by treatment with CPS1-IT1 siRNA **(C, D)**. p < 0.01 (**), p < 0.001 (***). **(E)** Human serum melatonin levels and hepatic CPS1-IT1 levels were analyzed using ELISA and real-time RT-PCR, respectively (n = 40). The CPS1-IT1 expression levels were positively correlated with the serum melatonin levels. **(F, G)** The inhibitory effects of melatonin on the proliferation of Huh7 and HepG2 cells were significantly reversed by treatment of these cells with CPS1-IT1 siRNA.

Recent studies have shown that lncRNAs mediate multiple cellular processes, including chromosome modification, transcription, translation, and protein activation. To determine whether lncRNAs are involved in the tumor suppressive effects of melatonin in the context of HCC, we analyzed the genome expression profiles of melatonin-treated Huh7 and HepG2 cells using whole-transcriptome sequencing analysis. We observed that the lncRNA expression profile of melatonin-treated cells was significantly different from that of control cells. Up to 79 lncRNAs showed significant expression changes in both cell lines after melatonin treatment, which indicates that these lncRNAs are likely involved in the anti-cancer effects of melatonin. Among the lncRNAs in question, the tumor suppressor lncRNA CPS1-IT1 showed one of the most profound differences in expression between the vehicle- and melatonin-treated cells (2.32-fold increase in melatonin-treated vs. vehicle-treated cells). To confirm that melatonin affects CPS1-IT1 expression, we performed quantitative real-time RT-PCR, which showed that the CPS1-IT1 expression levels were significantly increased in Huh7 and HepG2 cells treated with melatonin for 24 hr compared to control cells (Figure 3A).

Based on the findings of our previous study which showed that CPS1-IT1 could inhibit HIF-1α activation (thereby suppressing EMT) [40], we conducted a HIF-1α activity assay to determine whether melatonin regulates HIF-1α activity via CPS1-IT1 upregulation. As shown in Figure 3B, HIF-1α activity was significantly decreased in melatonin-treated cells compared to control cells; however, the inhibitory effects of melatonin were suppressed when CPS1-IT1 expression was silenced by the corresponding siRNA (Figure 3C-3D). Furthermore, the inhibitory effects of melatonin on cell proliferation in both HCC cell lines were significantly reversed by treatment with CPS1-IT1 siRNA (Figure 3F-3G).

To determine whether a correlation exists between the melatonin concentration and CPS1-IT1 expression levels under human physiological conditions, we analyzed the levels of serum melatonin and hepatic CPS1-IT1. We observed that the CPS1-IT1 expression levels in hepatic tissues were positively correlated with serum melatonin levels (Figure 3E). These results indicated that melatonin suppressed HCC progression partially via CPS1-IT1-mediated HIF-1α inactivation.

### Melatonin upregulated lncRNA-CPS1-IT1 expression by inducing FOXA2 expression

**Figure 4:**
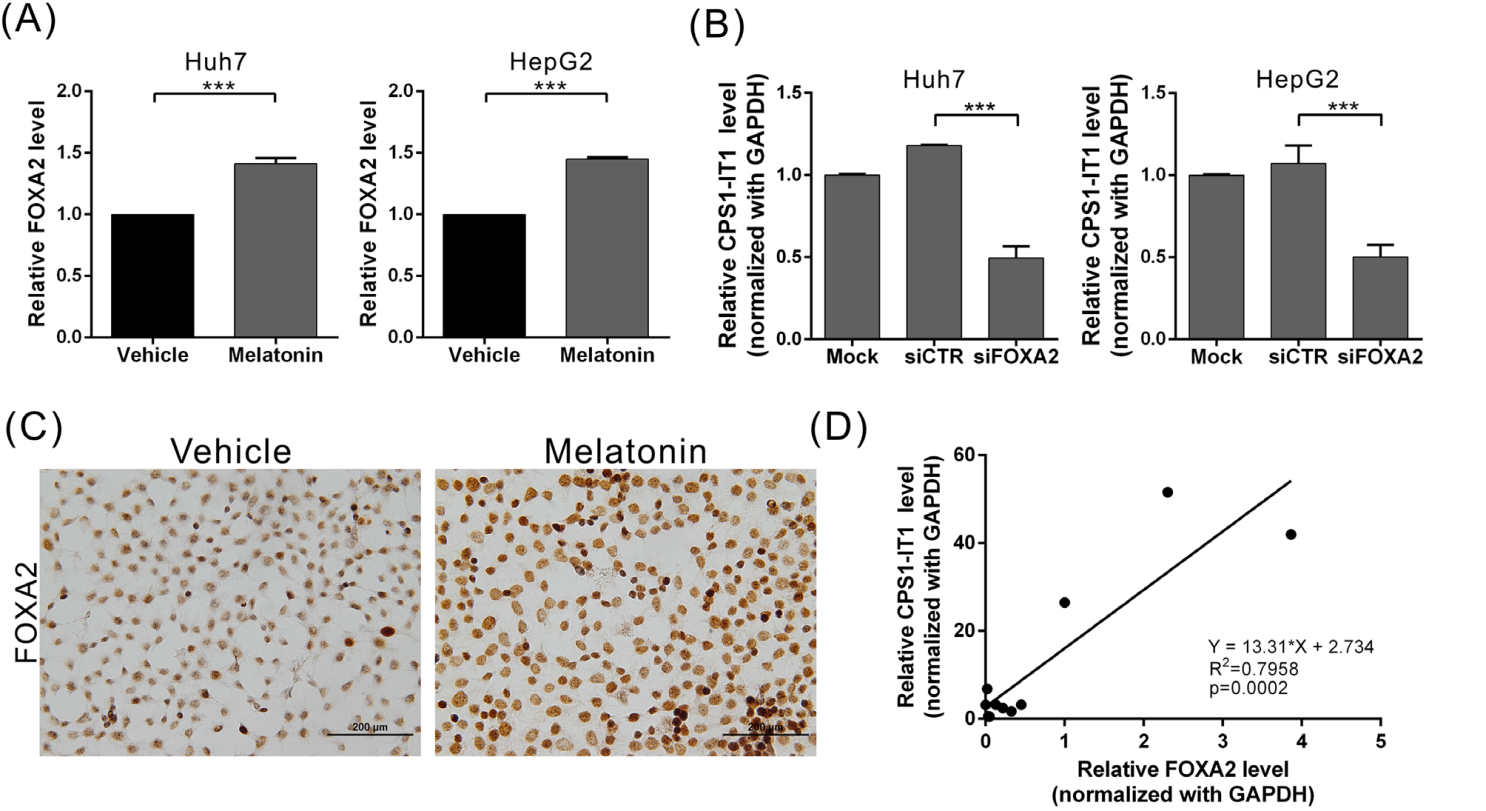
Melatonin induced lncRNA-CPS1-IT1 expression by upregulating FOXA2 expression **(A)** Huh7 and HepG2 cells were treated with melatonin for 24 hr, and FOXA2 expression was analyzed using quantitative real-time RT-PCR. **(B)** Silencing FOXA2 expression resulted in CPS1-IT1 downregulation in Huh7 and HepG2 cells. **(C)** Representative immunocytochemistry staining results show that melatonin upregulated FOXA2 expression in Huh7 cells. **(D)** The FOXA2 and CPS1-IT1 expression levels in human liver tissues were analyzed using real-time RT-PCR (n = 12). The CPS1-IT1 expression levels were positively correlated with the FOXA2 expression levels.

To determine which upstream regulatory factors regulate CPS1-IT1 expression, we performed promoter sequence analysis and transcription factor predictions using DBD transcription factor prediction database v2.0 software. The promoter region of CPS1-IT1 has a potential evolutionarily conserved binding site for FOXA2. To determine whether melatonin regulates FOXA2 expression, we analyzed the FOXA2 expression levels at 24 hr after melatonin treatment using quantitative real-time RT-PCR. We found that the FOXA2 expression levels were significantly increased in melatonin-treated Huh7 and HepG2 cells compared to control cells (Figure 4A). We observed similar results in the immunocytochemical staining analysis, which showed that melatonin upregulated FOXA2 expression levels in Huh7 cells (Figure 4C). To determine whether FOXA2 regulates CPS1-IT1 expression levels, we silenced endogenous FOXA2 expression and observed that the CPS1-IT levels were decreased in siRNA-treated cells compared to control cells (Figure 4B). Furthermore, we noted a positive correlation between the CPS1-IT1 expression levels and FOXA2 expression levels in human liver tissues (Figure 4D). These findings suggest that melatonin upregulates CPS1-IT1 expression by inducing FOXA2 expression.

### Melatonin suppressed tumor growth *in vivo*

**Figure 5:**
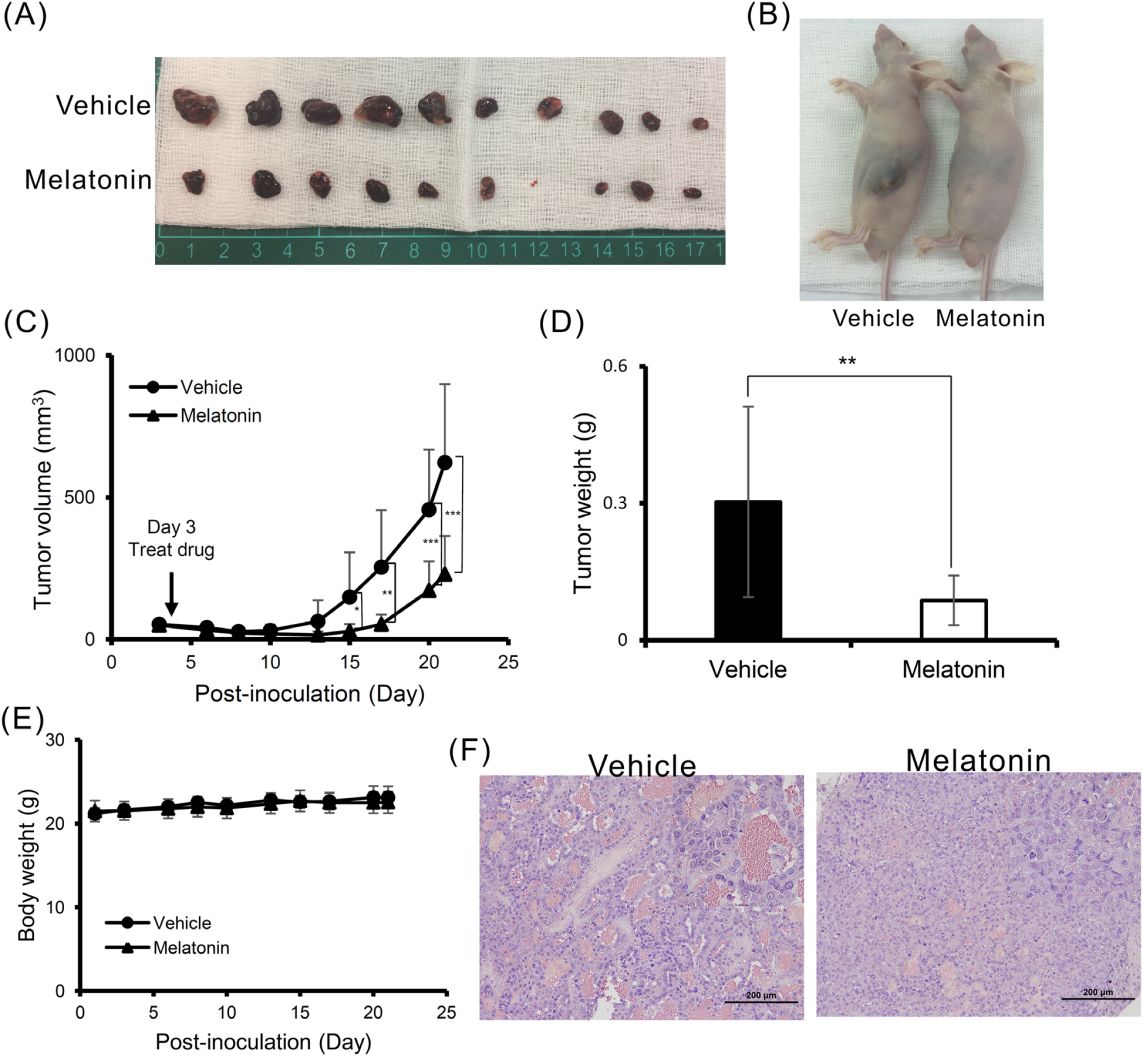
Melatonin suppressed tumor growth *in vivo* **(A, B)** A total of 5×10^6^ Huh7 cells were inoculated into nude mice (n = 10). Representative images show the tumor xenografts at 4 weeks after implantation. Melatonin treatment significantly reduced tumor growth. **(C)** The tumor volumes were calculated every 3 days after injection. The volume of each tumor was calculated as follows: length × width^2^ × 0.5. Bars indicate S.D. * p < 0.05, ** p < 0.01, *** p < 0.001. **(D)** Tumor weights are represented as the mean tumor weight ± S.D. **(E)** The body weight was measured every 3 days after injection. There was no significant difference in the body weight between the melatonin-treated and control mouse groups. **(F)** Histological analysis of the xenografts using H&E staining showed that melatonin promoted tumor cell differentiation. Magnification: 200×. Mean ± S.D. is shown.

To validate the tumor suppressive effects of melatonin, we generated an *in vivo* xenograft model. Consistent with our previous findings, melatonin inhibited HCC cell proliferation in a nude mouse model of tumor growth. At 28 days after melatonin injection, tumor growth was significantly reduced in the melatonin-treated group compared to the control group (Figure 5A-5D). Furthermore, we noted no significant difference in mouse body weight between the melatonin-treated and control groups, suggesting that melatonin does not exert toxic effects (Figure 5E). Additional hematoxylin and eosin (H&E) staining histologically showed that melatonin promoted tumor cell differentiation (Figure 5F).

**Figure 6:**
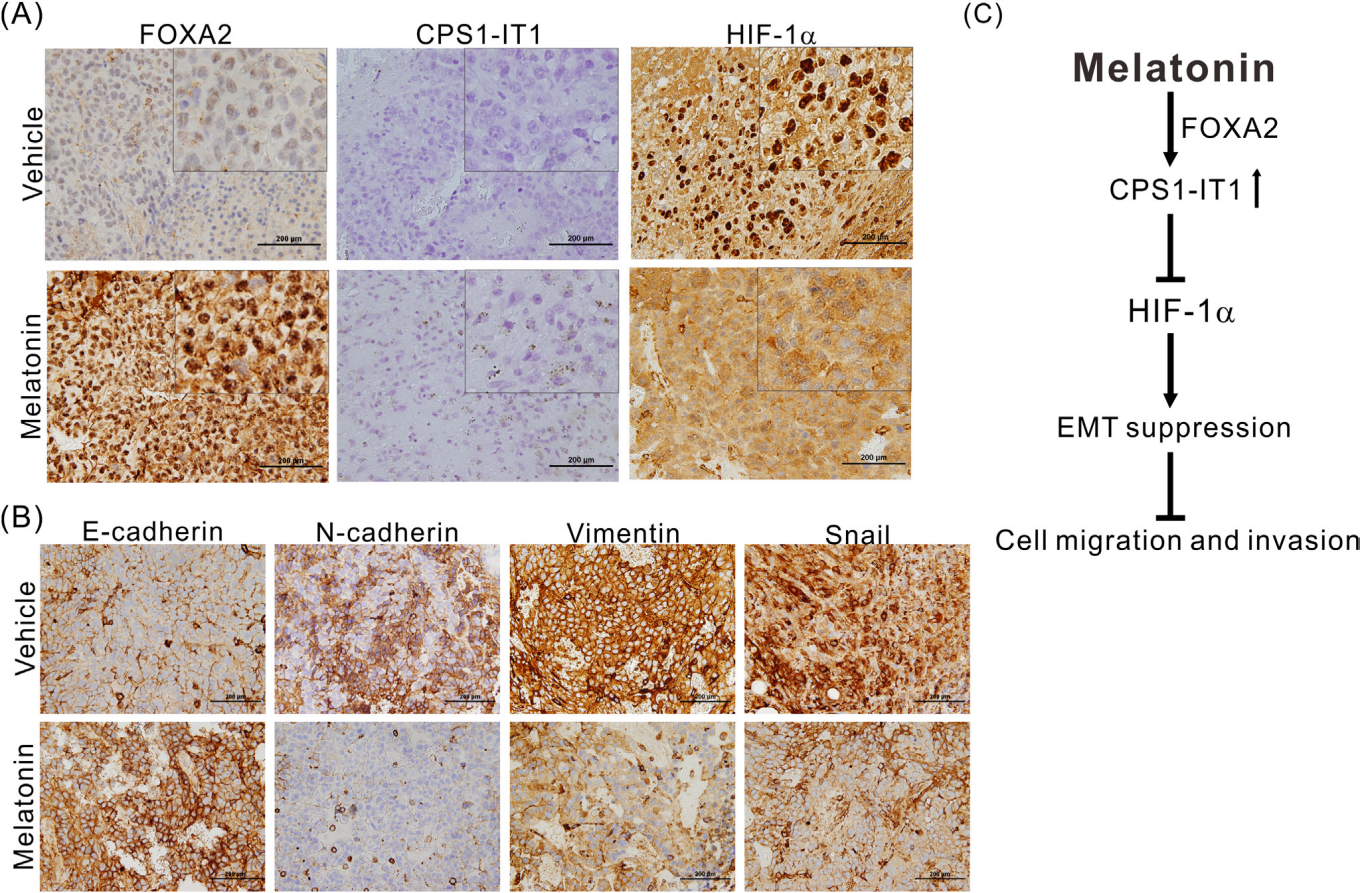
**(A)** Representative results of the *in situ* hybridization study of lncRNA-CPS1-IT1 and the immunohistochemical analysis of FOXA2 and HIF-1α expression levels in the xenografts of mice treated with either melatonin or vehicle. Melatonin induced FOXA2 and CPS1-IT1 expression and inhibited HIF-1α nuclear translocation. **(B)** Melatonin reduced the expression of EMT-related proteins as determined using immunohistochemical staining. **(C)** A schematic representation summarizing the anti-HCC mechanism of melatonin.

We also performed immunohistochemical staining and *in situ* hybridization to confirm that melatonin regulates EMT. As shown in Figure 6A, melatonin significantly induced FOXA2 and CPS1-IT1 expression and inhibited HIF-1α activation and its consequent nuclear translocation. Furthermore, similar to the results of the *in vitro* cell assays, the results of these experiments showed that compared to the control tumors, the melatonin-treated tumors exhibited downregulated expression of EMT-promoting proteins such as vimentin, N-cadherin and Snail as well as upregulated expression levels of E-cadherin (Figure 6B).

## DISCUSSION

To date, studies on melatonin have focused primarily on breast, lung, and colorectal cancer [41–46]. Only a few studies have investigated the effects of melatonin treatment on HCC. It has been reported that melatonin may inhibit cellular growth in HepG2 hepatoma cells and may enhance the sensitivity of these cells to radiation therapy [47–49]; however, the studies reporting these findings were *in vitro* studies that did not elucidate the molecular mechanisms underlying the effects of melatonin. In this study, we demonstrated the anti-HCC capacity of melatonin *in vitro* and *in vivo*. Melatonin upregulated lncRNA-CPS1-IT1 expression by inducing the expression of the transcription factor FOXA2 thereby promoting HIF-1α inactivation. These changes resulted in the suppression of EMT progression and HCC metastasis (Figure 6C). To the best of our knowledge, this is the first study to report that melatonin exerts anti-HCC activities by regulating lncRNA-CPS1-IT1 expression.

CPS1-IT1 is a tumor suppressor lncRNA that was initially identified in our previous study [40]. CPS1-IT1 can associate with Hsp90 and negatively modulate the binding affinity of Hsp90 for HIF-1α. The activity of several Hsp90 proteins, including HIF-1α, α-SMA and p-p38, all of which are important modulators in critical physiological processes such as cell proliferation, migration and invasion [50], is also modulated by CPS1-IT1, which may explain why melatonin is involved in regulating a diverse range of cellular physiological processes (including aging) and exerts anti-cancer effects. In this study, we demonstrated the existence of a regulatory network governing melatonin and the expression of FOXA2 and CPS1-IT1. Decreases in CPS1-IT1 expression noted in this study may be attributed to downregulation of FOXA2 in HCC tissues.

Endogenous melatonin exerts its physiological effects through the pharmacologically specific, high-affinity receptors MT1 (encoded by *MTNR1A*) and MT2 (encoded by *MTNR1B*), which are members of the G protein-coupled receptor superfamily [51]. However, the detailed mechanism by which melatonin regulates FOXA2 expression remains unclear. FOXA2 expression in cancer cells has been reported to be regulated by several mechanisms, including epigenetic phenomena [52, 53]. In this study, we analyzed the CpG island methylation of the FOXA2 promoter region but did not observe significant epigenetic modifications in melatonin-treated cells compared to control cells (data not shown). The precise mechanism by which melatonin induces FOXA2 expression requires further investigation.

HCC is the primary life-threatening malignancy of both sexes in Taiwan. However, as the rate of multidrug resistance in HCC is high, only 30% of affected patients respond to the popular drug sorafenib [54, 55]. Thus, it is important to explore novel strategies for diagnosing and treating HCC. In this study, we verified that melatonin exerts tumor suppressive effects against HCC and regulates the expression of lncRNA-CPS1-IT1 and its downstream genes. In addition, we also assessed the feasibility of melatonin administration as a treatment for HCC by performing *in vivo* animal experiments. Understanding the mechanisms by which melatonin exerts its effects is imperative for both assessing the feasibility of its use as a treatment for HCC and providing clinicians with a reference to identify patients who are most suitable for melatonin treatment alone and those who are most suitable for a combination therapy comprising melatonin and other anti-cancer drugs. Taken together, our findings suggest that melatonin inhibited HCC progression by modulating lncRNA-CPS1-IT1-mediated EMT suppression and support the idea that melatonin has potential as a therapy for HCC.

## MATERIALS AND METHODS

### Measurement of melatonin, FOXA2 and CPS1 intronic transcript 1 (CPS1-IT1) levels in human specimens

Serum samples and fresh frozen tissue samples from forty patients with HCC were obtained from the tissue bank at Lin-Kou Chang Gung Memorial Hospital. Each patient provided written informed consent prior to participation in this study. The serum melatonin levels were measured using an enzyme-linked immunosorbent assay (ELISA) kit (CUSABIO, Baltimore, MD, USA), according to the manufacturer’s instructions, and FOXA2 and CPS1-IT1 expression levels in the collected tissue samples were analyzed using quantitative real-time RT-PCR with a TaqMan gene expression assay (cat. no. 4331182, Thermo Fisher Scientific, MA, USA). This study was approved by the Ethics Committee of Chang Gung Memorial Hospital.

### Cell lines, antibodies, siRNAs and plasmid construction

The HCC cell lines HepG2 and Huh7 were obtained from the American Type Culture Collection (Manassas, VA, USA) and cultured in DMEM supplemented with 10% fetal bovine serum at 37°C in an atmosphere containing 5% CO_2_ atmosphere. Antibodies against E-cadherin (24E10), N-cadherin, claudin-1 (D5H1D) and Snail (C15D3) were purchased from Cell Signaling Technology (Beverly, MA). Antibodies against HIF-1α (GTX 127309), FOXA2 (GTX 84485), vimentin (GTX 100619) and Slug (GTX 121924) were purchased from GeneTex (Irvine, CA). Antibodies against β-actin were purchased from Sigma (St. Louis, MO, USA), and appropriate secondary antibodies were purchased from Santa Cruz Biotechnology (Santa Cruz, CA). A commercially produced si-CPS1-IT1 (cat. no. 4390771) and negative control siRNA (cat. no. 4464058) were purchased from Thermo Fisher Scientific.

### Whole transcriptome library preparation and sequencing

Total RNAs from HCC cells treated with and without melatonin were isolated by TRIzol reagent (Invitrogen, Carlsbad, CA) and quality was evaluated using the Bioanalyzer 2100 system (Agilent Technologies, Santa Clara, CA, USA). Ribosomal RNA (rRNA) was removed with the Epicentre Ribo-zero rRNA Removal Kit (Epicentre, Madison, WI, USA) and libraries were generated using the rRNA-depleted RNA by NEBNext Ultra Directional RNA Library Prep Kit for Illumina (NEB, Ipswich, MA, USA) following the manufacturer’s recommendations. First-strand cDNA was synthesized using random hexamer primer and M-MuLV Reverse Transcriptase. Second-strand cDNA synthesis was performed with DNA polymerase I and RNase H. To select cDNA fragments 150–200 base pairs in length, library fragments were purified with the AMPure XP system (Beckman Coulter, Brea, CA, USA) after adenylation of 3′ ends of DNA fragments and ligation of the NEBNext Adaptor. Sequencing was performed on an Illumina Hiseq 2000 platform and paired-end reads of 100 base pairs were produced according to Illumina’s protocol. Approximately 12 gigabases of cDNA sequence per sample were generated for subsequent data analysis.

### RNA-Seq data analysis

After removing adapter sequences from the raw data, the individual library was converted to FASTQ format. Sequence reads were aligned to the human genome (hg19) with TopHat2 [56], and the resulting alignment data were reconstructed with Cufflinks [57] and Scripture [58]. The RefSeq database (Build 37.3) and GENCODE v19 database were used as annotation references for mRNA and lncRNA analyses, respectively. Protein coding potential of transcripts was further analyzed by Coding Potential Calculator [59], Pfam-scan [60], phylogenetic codon substitution frequency [61], and Coding-Non-Coding-Index [62]. Transcript assembly, abundance estimates, and differential expression analyses were conducted by using Cufflinks2 and Cuffdiff2 [56, 63]. Biological sample gene variance was not determined because differential gene expression comparison was run without biological replicates. Gene or exon expression levels were normalized to the number of reads per kilobase per million mapped reads [64]. An adjusted *p* value < 0.05 (Student’s *t*-test) was used as the cut-off for determining differentially expressed genes.

### Detection of FOXA2 and lncRNA-CPS1-IT1 expression using quantitative real-time RT-PCR

Total RNA was isolated from each tissue sample using an RNeasy mini kit (QIAGEN, Gaithersburg, MD, USA) according to the manufacturer’s instructions. Two micrograms of RNA were reverse transcribed to generate cDNA, which was subsequently subjected to quantitative real-time RT-PCR using a TaqMan gene expression assay (Applied Biosystems, Foster City, CA) to detect FOXA2 and lncRNA-CPS1-IT1 expression. GAPDH was used as an internal control.

### Detection of protein expression levels using western blot analysis

Cells treated with 1 mM melatonin for 24 and 48 hr were harvested, washed twice with phosphate-buffered saline (PBS), and lysed in 200 μl of RIPA lysis buffer (50 mM Tris–HCl, pH 7.4; 150 mM NaCl; 1 mM EDTA, 1% Triton X-100; 1% sodium deoxycholate; 0.1% SDS) containing protease inhibitors. Equal amounts of protein (100 μg) from the supernatant of each sample were loaded onto an SDS polyacrylamide gel, and western blot analysis was performed to detect the expression levels of the proteins in response to melatonin. The immunoreactive bands were visualized using an ECL system (NEN Life Science Products, Boston, MA) and developed using X-ray films. The density of each band was quantified using ImageQuant 5.2 software (GE Healthcare, Piscataway, NJ).

### Immunocytochemistry

A total of 1×10^4^ cells were cultured per slide and treated with either 1 mM melatonin or vehicle. After a 48-hr incubation, cells were harvested and fixed in 100% methanol for 10 min. The slides were washed twice with 1× PBS, blocked with 10% horse serum, and incubated with FOXA2 antibodies. After the slides were rinsed three times with 1× PBS, they were treated with horseradish peroxidase conjugated secondary antibodies, after which the cells were chromogenized and counterstained with hematoxylin.

### HIF-1α activity assay

HIF-1α activity was analyzed using a Cignal HIF Reporter assay (Qiagen, Hilden, Germany). Cell lines (3×10^5^ cells/well) were seeded in 6-well plates and incubated overnight. The cells were then treated with either 1 mM melatonin or vehicle. At 12 hr after treatment, the cells were transfected with 1 μg of HIF-1 reporter mixture. Afte an additional 48 hr, the cells were harvested and subjected to a luciferase assay.

### Cell proliferation assay

The proliferative capacity of the cells was monitored using an xCELLigence real-time cell analyzer (Roche Life Science, Indiana, USA) and examined via colony formation assay according to the manufacturer’s instructions. For the colony formation assay, cells were seeded onto 6-well plates at a density of 500 cells/well and maintained in DMEM containing either 1 mM melatonin or vehicle for 12 days. The medium was replaced every 3 days. The resulting colonies were subsequently fixed with methanol and stained with 0.1% crystal violet (Sigma-Aldrich, St. Louis, MO, USA). Visible colonies were imaged and manually quantified.

### Cell migration and invasion assay

Cell migration was analyzed via wound-healing and transwell migration assays as previously described [32]. For the wound-healing assay, Huh7 and HepG2 cells were plated onto 6-well plates and cultured to 90% confluence. The cells were then scraped with a 200 μl pipette tip (time 0), and the medium was replaced with low-serum culture medium containing either 1 mM melatonin or vehicle. The distances the cells migrated were measured based on images (five randomly selected fields) taken at the indicated time points.

The migratory and invasive abilities of Huh7 and HepG2 cells were assessed using ThinCert Tissue Cell Culture Inserts (Greiner Bio-One, Kremsmunster, Austria) containing a membrane with 8-μm pores. For the migration assay, cells were trypsinized and resuspended in serum-free culture medium (DMEM) at a density of 5×10^5^ cells/ml. The lower chambers were filled with 500 μl of complete medium (DMEM supplemented with 10% FBS), and 100-μl aliquots of the cell suspension were added into each upper well. The chambers were then incubated for 24 hr at 37°C in a humidified atmosphere containing 5% CO_2_. Afterward, the cells were subsequently fixed with 500 μl of methanol for 15 min, and the cells remaining on the inner surfaces of the upper chambers were removed using cotton swabs to wipe away the non-migratory cells. The membrane was then washed with 500 μl of PBS and stained with 500 μl of crystal violet for 20 min at room temperature. After the cells were again washed with 500 μl of PBS, they were imaged using ImagePro 6.2 software. Counts were performed in five random fields at 100× magnification. For the invasion assay, the procedure was performed as described for the transwell migration assay with two exceptions: 1) the membranes were coated with 30 mg/cm^2^ Matrigel (ECM gel, Sigma-Aldrich, St. Louis, MO) to form a matrix barrier, and 2) the invasion time was 48 hr instead of 24 hr.

### Mice

Six- to eight-week-old male nude mice (BALB/cAnN-Foxnlnu/CrlNarl mice, purchased from the National Laboratory Animal Center, Taipei, Taiwan) were housed under pathogen-free conditions and a 12 hr light/12 hr dark cycle and provided autoclaved standard chow and water. The mice were bred at the animal center of Chang Gung Hospital (Tao-yuan, Taiwan) according to the Guidelines for the Care and Use of Laboratory Animals established by the NIH. All the animal experiments were approved by the Institutional Animal Care and Use Committee (IACUC) at Chang Gung Hospital.

### Xenograft assays and melatonin administration

Mice were anesthetized with a xylazine (100 mg/kg, i.p.) and ketamine (10 mg/kg, i.p.) cocktail to chemically restrain the animals. A cell suspension of 5×10^6^ Huh7 cells in 100 μl of 50% Matrigel (BD Biosciences) in saline was subcutaneously implanted into the left and right flank regions of the anesthetized mice. All the tumors were staged for 1 week before drug treatments were initiated. At the beginning of the second week, the mice with tumors were intraperitoneally (IP) injected five days per week with 100 μl of either melatonin (at dose of 40 mg/kg of body weight) or dimethyl sulfoxide (DMSO), which served as a control. Melatonin was administered 1 hr before the room lights were switched off. The tumor volumes were measured three times per week using digital calipers.

### Immunohistochemistry

The slides used for the immunohistochemical analysis were initially incubated for 30 min at 65°C followed by deparaffinization in xylene and rehydration in graded ethanol solutions. Afterward, the slides were boiled in Trilogy reagent (Cell Marque, Rocklin, CA) for 10 min in a microwave oven for antigen retrieval. The slides were washed with 1× PBS and were immersed in a 3% hydrogen peroxide solution for 10 min to suppress endogenous peroxidase activity. After the slides were rinsed three times with 1× PBS, the sections were incubated with the appropriate primary antibodies for 1 hr at room temperature, rinsed three times with 1× PBS, and incubated with a biotinylated secondary antibody (Dako, Glostrup, Denmark) for 25 min. The slides were rinsed three times again with 1× PBS and treated with horseradish peroxidase-conjugated streptavidin for 25 min at room temperature. Peroxidase activity was detected using DAB substrate (Dako), which served as a chromogen, at room temperature, after which the slides were counterstained with hematoxylin.

### LncRNA *in situ* hybridization

The expression and localization of the lncRNA CPS1-IT1 in tissues were analyzed with custom-made probes using an RNAscope 2.0 FFPE Assay-Brown kit according to the manufacturer’s instructions *(Advanced Cell Diagnostics*, Inc., Hayward, CA). Briefly, paraffinized sections were incubated at 60°C for 1 hr and deparaffinized with xylene and 100% ethanol. After this pretreatment, the sections were hybridized to target probes for 18 hr at 40°C. The slides were then sequentially treated with Amp1 (preamplifier), Amp2 (signal enhancer), Amp3 (amplifier), Amp4 (probe labeler), Amp5 (signal amplifier) and Amp6 (HRP-linked labeling molecule). After these amplification steps were completed, the DAB substrate was added for the colorimetric detection of the target RNA. Finally, the slides were counterstained with hematoxylin (Sigma-Aldrich, St. Louis, MO, USA) and mounted using whole-mount medium.

### Data analysis

The original real-time PCR, western blotting and migration assay data were recorded as continuous variables and were analyzed using Student’s *t*-test. All statistical analyses were performed using SPSS 16.0 and Excel 2007 software. All statistical tests were two-sided, and *P* values < 0.05 (*), < 0.01 (**), or < 0.001 (***) were considered statistically significant. The presented results were representative of three independent experiments with similar results.
